# Demographics and clinical features of elderly patients undergoing regular dialysis in Brazil

**DOI:** 10.1590/1414-431X20209806

**Published:** 2021-02-12

**Authors:** J.G. Gonçalves, J.R. Lugon, M.M. do Nascimento, R.C. Sesso

**Affiliations:** 1 Departamento de Medicina, Divisão de Nefrologia, Faculdade de Medicina, Universidade de São Paulo, São Paulo, SP, Brasil; 2 Departamento de Medicina, Divisão de Nefrologia, Faculdade de Medicina, Universidade Federal Fluminense, Niterói, RJ, Brasil; 3 Departamento de Medicina, Divisão de Nefrologia, Faculdade de Medicina, Universidade Federal do Paraná, Curitiba, PR, Brasil; 4 Departamento de Medicina, Divisão de Nefrologia, Faculdade de Medicina, Universidade Federal de São Paulo, São Paulo, SP, Brasil

**Keywords:** Chronic kidney failure, Aging, Renal dialysis, Epidemiology

## Abstract

An increasing number of elderly people in renal support is expected in the coming years. The objective of this study was to report the clinical and socio-demographic data of end-stage renal disease (ESRD) adult patients undergoing regular dialysis treatment comparing elderly (≥65 years old) and non-elderly subjects using data from the Brazilian Dialysis Registry database. The regional distribution of the sample was Southeast (48.8%), South (33.7), Northeast (13.1%), Midwest (5.1%), and North (0.1%). A total of 18,030 patients were included in the analysis with elderly patients accounting for 29.5% of the sample. The elderly patients were predominantly male, white, retired, and literate. Elderly ESRD patients had a slightly higher frequency of undernourishment and a lower frequency of obesity than the non-elderly adults. A higher frequency of elderly patients were from the South and Southeast regions. The dialysis treatment of patients from both groups was predominantly funded by the public system, but the percent of non-public funding was higher for the elderly group. The most used initial access in the elderly was the central venous catheter and hemodialysis was the main modality at the beginning of treatment (93.2%), as well as during maintenance therapy (91.8%). Advanced age was associated with greater use of central venous catheter in the first dialysis session. The survival of the elderly on dialysis was lower than that of the non-elderly early in the course of dialysis and this difference increased over time. This is yet the largest national epidemiological study of elderly people on chronic dialysis.

## Introduction

In most countries, the prevalence of end-stage renal disease (ESRD) patients has been increasing in recent years (1–3). This may be accounted for by population aging and poor socioeconomic conditions, as well as by an increase in the incidence of diabetes and hypertension, the two major causes of ESRD worldwide. In addition, improvements in the diagnosis of chronic kidney disease due to increased medical and patient awareness about the disease may also have contributed to this increase (4).

Age alone does not exclude the indication of dialytic support. The aging of the population coupled with improved management of comorbidities, technological advances in renal replacement therapy (RRT), more liberal admittance to dialysis, and better acceptance of dialysis support have resulted in the expansion of the geriatric population on dialysis. This increase has been particularly seen in the age group above 75 years, who may carry multiple comorbidities and are less independent and more fragile (5,6). However, the prognosis and decision-making process regarding the start of chronic dialysis in this age group remains controversial (6).

Worldwide geographical, cultural, and funding inequities of this costly treatment are not properly reported in the literature with most epidemiological data regarding elderly ESRD patients coming from developed countries (5–7). In Brazil, it was estimated that over 125,000 people were undergoing dialysis by July 2017 (8), with an estimated annual expenditure of 2.2 billion Brazilian reals (9). We used data from the Brazilian Registry from 2011-2017 for an analysis of the panorama of elderly dialysis patients in Brazil.

## Material and Methods

In 2010, the Brazilian Society of Nephrology Working Committee for the Brazilian Dialysis Registry developed an electronic database for collecting clinical and epidemiological information from patients undergoing chronic dialysis therapy in the country. The largest companies that provide software for patients' management at dialysis centers all over the country were contacted. Several of the companies agreed to participate in this initiative and adapted their software system to conform to our standard registry framework developed by the study coordinating committee. The collected information comprised socio-demographic data, clinical characteristics, primary renal diagnosis, characteristics of dialysis therapy, comorbidities, routine laboratory tests and medications, last patients' follow-up information, and data concerning being alive on therapy, dead, or having received a renal transplant. The participating companies were Nefrodata^©^, Hemosys^©^, Nefrosoft^©^, Nefrosys^©^, Dialsist^©^, and Fresenius^©^ (7).

Since 2011, the Coordinating Committee of the Brazilian Dialysis Registry has accepted the inclusion of units that volunteer to submit data for registration. For the analysis performed in this study, we used data collected from January 2011 to July 2017, encompassing 73 participating centers. The regional distribution of these units maintained a proportionality between the active centers by region of the country, although there is a predominance of representation of the southern states and negligible participation of the northern region. The regional distribution observed was Southeast (48.8%), South (33.7), Northeast (13.1%), Midwest (5.1%), and North (0.1%). The Institutional Review Committee of the Federal University of São Paulo approved the study and confidentiality of all patient information in the registry was guaranteed (7).

Patients who initiated dialysis before inclusion in the Registry configured prevalent cases and those whose data collection was ongoing, incident cases. Data are mainly reported in descriptive form. The comparisons of categorical variables between the age groups were verified using the chi-squared test, and means were compared by the Student's *t*-test. Multiple logistic regression analysis was used to evaluate the effect of age group on the use of a central venous catheter as the first vascular access at the time of the onset of hemodialysis, while simultaneously adjusting for sociodemographic characteristics and primary renal disease. The variables tested in the multivariate model were those with results significantly different in the univariate analyses comparing the ≥65-year and 18- to 64-year groups. Gender was included in this analysis due to its clinical relevance. We opted *a priori* to assess the effect of age group on catheter use in a fully adjusted model, entering all tested variables in the model. The significance level was established at P<0.05.

Survival curves using the Kaplan Meier method were calculated for the incident group and factored by age. The timeline began at the date of commencement of renal replacement therapy and ended at the date of death, kidney transplantation, or end of follow-up, whichever came first.

## Results

Of the 24,930 patients enrolled during the period, 18,030 had age information at the beginning of dialysis and were included in the study. Patients were subdivided by age in elderly (65 years or older; n=5,324; 29.5%) and non-elderly (18 to 64 years old; n=12,706; 70.5%) patients. As shown in Table 1, the elderly ESRD patients were preponderantly male, white, retired, and literate (although mostly with elementary and middle school education level). The percent of people of white skin color and retired was significantly higher in the elderly whereas the percent of illiterate people was lower compared with the non-elderly adults. Elderly ESRD patients had a slightly higher frequency of undernourishment and a lower frequency of obesity than the non-elderly adults. The majority of the patients of the study sample came from the South and Southeast regions of the country and the frequency of patients from these two regions was higher in the elderly patients compared with the non-elderly adults.

**Table 1:** Sociodemographic characteristics of dialysis patients by age group.

	≥65 years	18-64 years	P value
Gender (n=18,030)			
Male	3,125 (58.7)	7,349 (57.8)	0.290
Female	2,199 (41.3)	5,357 (42.2)	
Skin color (n=17,942)			
White	3,671 (69)	6,932 (55.0)	<0.001
Non-white	1,626 (31)	5,713 (45.0)	
Education level (n=11,775)			
Illiterate	557 (16.9)	941 (11.1)	<0.001
Elementary and middle school	2,002 (60.7)	4,917 (58.0)	
High School	462 (14)	1,907 (22.5)	
Higher education	278 (8.4)	711 (8.4)	
Employment (n=7,662)			
Partial time	284 (11.8)	999 (19.0)	<0.001
Housewife/husband	201 (8.4)	489 (9.3)	
Retired	1,862 (77.6)	3,356 (63.8)	
Other	54 (2.2)	417 (7.9)	
Body mass index (n=14,009), kg/m^2^			
<18.5	374 (9.8)	812 (8.0)	<0.001
18.5-25	1,956 (51.2)	5,110 (50.1)	
>25 to ≤30	1,076 (28.20)	2,773 (27.2)	
>30 to ≤40	395 (10.3)	1,379 (13.5)	
>40	17 (0.4)	117 (1.1)	
Regions of the country (n=17,650)			
South	1,902 (36.3)	4,042 (32.6)	<0.001
Southeast	2,555 (48.8)	5,913 (47.6)	
Midwest	248 (4.7)	657 (5.3)	
Northeast	526 (10)	1,793 (14.4)	
North	4 (0.1)	10 (0.1)	

Data are reported as frequency and percentages. n: number of valid answers for the variable. Chi-squared test.

Table 2 shows some selected characteristics of dialysis treatment. The percent of incident cases among the elderly population was higher than in non-elderly ones. The mean age at onset of dialysis in elderly patients was 73.4±6.5 years. The dialysis treatment of patients from both groups was predominantly funded by the public health system, but the percent of non-public funding was higher for the elderly ones. A higher percentage of elderly patients underwent conservative treatment before dialysis onset than younger patients (55.4 *vs* 46.5%, P<0.001). Most patients in both age groups started dialysis therapy in an outpatient clinic instead of in a hospital. The vast majority of patients underwent hemodialysis as the initial RRT modality, however, the elderly had a slightly higher but significant frequency of peritoneal dialysis. A similar framework was seen when the current dialysis modality was analyzed. The most commonly used initial vascular access in both groups was a central venous catheter (CVC), but the frequency of its use was significantly higher in the elderly compared with the 18-64 age group. Diabetes kidney disease and hypertensive nephropathy were the primary renal diseases in both groups but the frequency of each one of these disorders as the cause of ESRD was higher in elderly patients. The frequency of lupus nephropathy as the cause of ESRD in the elderly group was substantially lower.

**Table 2:** Selected characteristics of the dialysis treatment by age group.

	≥65 years	18-64 years	P value
Type of patient (n=18,048)			
Prevalent	2,415 (45.3)	8,150 (64.1)	<0.001
Incident	2,916 (54.7)	4,567 (35.9)	
Age at dialysis start (n=18,048), years	73.4±6.5	47.1±12.1	<0.001
Funding (n=17,949)			
Public	3,632 (68.5)	10,402 (82.2)	<0.001
Health Insurance Co.	1,668 (31.5)	2,247 (17.8)	
Conservative treatment before dialysis beginning (n=5,104)	829 (55.4)	1,676 (46.5)	<0.001
Place of initial dialysis (n=6,598)			
Hospital	810 (42.3)	1,897 (40.5)	0.091
Outpatient clinic	1,104 (57.3)	2,787 (59.5)	
First dialysis modality (n=8,890)			
Hemodialysis	2,317 (93.2)	6,084 (95)	0.001
Peritoneal dialysis	168 (6.8)	489 (5.5)	
First vascular access (n=6,200)			
Primary A-V fistula	517 (29.3)	1,663 (37.5)	<0.001
Graft A-V fistula	7 (0.4)	7 (0.2)	
Central venous catheter	1,243 (70.3)	2,763 (62.3)	
Current dialysis modality (n=16,951)			
Hemodialysis	4,536 (91.8)	11,147 (92.8)	0.031
Peritoneal dialysis	403 (8.2)	865 (7.2)	
Primary renal disease (n=15,412)			
Diabetes kidney disease	1,026 (23.1)	2,138 (19.5)	<0.001
Hypertensive nephropathy	1,009 (22.8)	2,115 (19.3)	
Chronic glomerulonephritis	85 (1.9)	1,119 (10.2)	
Adult polycystic kidneys	103 (2.3)	463 (4.2)	
Interstitial nephritis	73 (1.6)	209 (1.9)	
Lupus nephropathy	3 (0.1)	86 (0.8)	
Myeloma kidney disease	24 (0.5)	28 (0.3)	
Other	1,180 (26.6)	2,975 (27.1)	
Undetermined	927 (20.9)	1,849 (16.8)	

Data are reported as frequency and percentages or mean±SD. n: number of valid answers for the variable; A-V: arteriovenous. Chi-squared test and Student’s t-test.

Table 3 shows a multivariate logistic regression analysis model to test for associations with CVC use as the first vascular access for chronic hemodialysis. The variable of primary interest (age ≥65 years) was associated with a 35% greater chance of CVC use. In addition, having private health insurance increased the chance of CVC use by 24%. Taking the Northeast region as reference, treatment in the Midwest region increased 3.8 times the chance of CVC use, whereas treatment in the Southeast region reduced such chance by 58%. Having diabetes kidney disease or hypertensive nephropathy as the cause of ESRD also increased the chance of CVC use (by 38, 24%, respectively).

**Table 3:** Multivariate logistic multiple regression model to test for association with the use of a central venous catheter as the vascular access at the time of the onset of dialysis.

	OR (95%CI)	P value
Age, ≥65 years	1.35 (1.14-1.60)	<0.001
Male gender	0.95 (0.83-1.10)	0.499
Dialysis funded by a health insurance company	1.24 (1.02-1.50)	0.031
White skin color	1.02 (0.88-1.19)	0.788
Body mass index, kg/m^2^	0.991 (0.98-1.00)	0.156
Region origin		
Northeast	1.00	-
South	1.12 (0.88-1.43)	0.356
Southeast	0.42 (0.33-0.53)	<0.001
Midwest	3.81 (2.42-6.0)	0.001
Primary renal disease		
Non-diabetic, non-hypertensive kidney disease	1.00	-
Hypertensive nephropathy	1.38 (1.15-1.65)	0.001
Diabetes kidney disease	1.24 (1.05-1.47)	0.013
Education level		
Higher education	1.00	-
Illiterate + incomplete elementary school	1.06 (0.78-1.42)	0.720
Elementary school + Middle school	1.12 (0.83-1.51)	0.468

**Figure 1:**
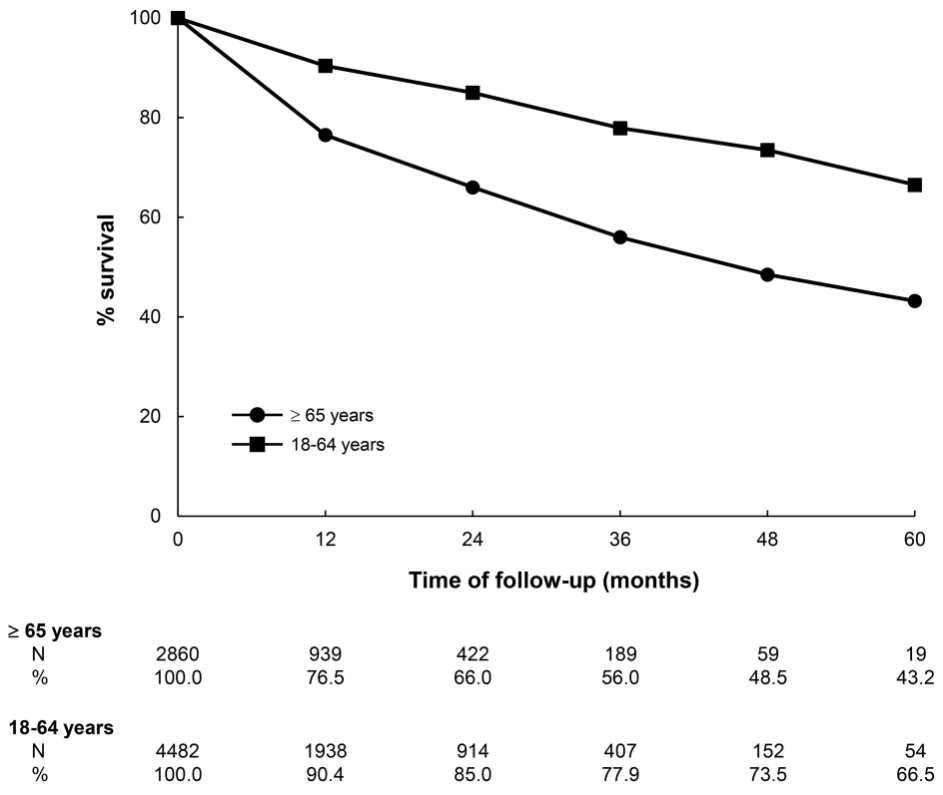
Survival curves of incident dialysis patients by age group. P<0.001, comparison between the curves.

Finally, Figure 1 shows that at the end of 5 years, the cumulative survival rate of elderly dialysis patients was significantly lower than that of non-elderly adults (43.2 *vs* 66.5%, P<0.001). Of note, the survival difference was already present in the first 6 months of follow-up.

## Discussion

Analysis of the data regarding the elderly population on chronic dialysis raises information that is important for the development of public health strategies. An increase in the number of elderly patients on renal support is expected in the coming years, which can lead to a burden for the health system due to the demands related to chronic kidney disease especially when accompanied by multiple comorbidities. Elderly people on dialysis also have high rates of mortality and frailty that demand social support, not always available (2).

The Brazilian Dialysis Registry is a reliable and robust database with large sampling and easy management. It should be stressed though, that adherence to questionnaires by clinics in the country is uneven, with a preponderant representation of dialysis centers in the southern region. The northern region, which has the lowest number of dialysis centers in the country (n=37), had negligible participation in this sample until the end of the study period. The database as a whole comprised a sample of about 25,000 patients and allowed us a consistent overview of the panorama of ESRD in the Brazilian context (7).

Similarly to epidemiological studies from Brazil (10,11) or different countries around the world (12), elderly patients were predominantly male (58.7%) and white (69.3%). As expected, the vast majority of renal support treatments in the elderly (68.5%) were funded by the Brazilian public health system.

The employment situation in this age group showed a very low percentage of people with active employment (11.8%), attributable to regular or disease-related retirement. Such specific questions, however, had a low response rate and their interpretation requires caution. Regarding educational status, only 16.9% of the elderly patients declared themselves illiterate.

Our findings regarding body mass index (BMI) in this population deserve attention. Historically, malnutrition has been a concern in the group of chronic renal patients. However, the numbers for BMI were within or above the reference values in 90% of the elderly patients. Together, overweight and obesity affected almost 40% of the elderly patients whereas undernourishment was restricted to ∼10% of cases. The findings are in agreement with data from developed countries (12,13) and reinforce the importance of prevention and treatment of overweight/obesity in ESRD patients.

Hemodialysis was the main modality at the beginning of treatment (93.2%) as well as for chronic maintenance therapy (91.8%) of the elderly group on renal support. These data were in agreement with those reported in most countries (7). Similar to reports from developed countries (12), diabetic nephropathy was the predominant primary kidney disease (23.1%) in elderly dialysis patients. However, caution should be exercised with these data due to the high percentage of undetermined diagnosis/others, which raises doubts regarding the quality of information yielded by the dialysis centers.

In the multivariate analysis, older age remained a significant factor associated with a higher frequency of use of CVC in the first dialysis session, even after the adjustment for several socio-demographic variables, underlying disease, treatment region, and types of health insurance. Another important finding was the observation of a significantly lower cumulative survival rate of elderly patients in dialysis compared to non-elderly adults, evidenced a few months after the beginning of treatment. At five years, the survival rate was 23% lower compared to non-elderly adults, findings consistent with the literature (14).

Clinical guidelines recommend arterio-venous fistula (AVF) as the best initial access due to the lower complication rate and better prognosis compared to graft or venous catheter users. However, the degree of evidence used for such a recommendation is low (15). It is known from large observational studies, such as the data analysis of the DOPPS (14), that there is a higher mortality rate in CVC and graft users compared with AVF users. However, this result does not appear to be attributable to higher access complication rates in the first group. One possible explanation could be that most debilitated patients are more frequently assigned to CVC or graft as vascular access. Adequate patency of an AVF may take months or even not be achieved in older and sicker patients due to their compromised vasculature (14).

Another conceivable hypothesis is that elderly patients with advanced CKD (or their nephrologists) are reluctant of the idea of initiating dialysis in the near future. This mindset could lead to a delay in placing an AVF and, ultimately, to emergent initiation of dialysis via a CVC.

In summary, the purpose of this analysis was to offer an overview of elderly dialysis patients in Brazil and provide information that could help the development of public health strategies focused on this specific population. Our study had some limitations including irregular adherence to filling out epidemiological questionnaires, absence of a detailed analysis of comorbidities, and the scarcity of data regarding the population of the Northern region, although this part of the country only comprises 5% of dialysis patients of the country. Additionally, as the included dialysis units were not obtained randomly among the Brazilian centers, the generalization of the results to the whole country should be cautious. Although we do not have information about the percentages of CVCs that were tunneled or temporary, another national database showed that these figures are about 61 and 39%, respectively (8). This study does have strengths such as the large sample size, the wide variety of epidemiological aspects evaluated, and the prospective nature of the analysis of the incident patients. Further studies are needed to establish the proper time to start the replacement of the renal function and which modality of RRT is the best choice for the growing elderly population with ESRD.
